# Periodic Distribution of a Putative Nucleosome Positioning Motif in Human, Nonhuman Primates, and Archaea: Mutual Information Analysis

**DOI:** 10.1155/2013/963956

**Published:** 2013-06-10

**Authors:** Daniela Sosa, Pedro Miramontes, Wentian Li, Víctor Mireles, Juan R. Bobadilla, Marco V. José

**Affiliations:** ^1^Facultad de Ciencias, Universidad Nacional Autónoma de México, 04510 CP, DF, Mexico; ^2^Centro de Ciencias de la Complejidad, Universidad Nacional Autónoma de México, 04510 CP, DF, Mexico; ^3^The Robert S. Boas Center for Genomics and Human Genetics Manhasset, Feinstein Institute for Medical Research, North Shore LIJ Health System, Manhasset, NY, USA; ^4^Theoretical Biology Group, Instituto de Investigaciones Biomédicas, Universidad Nacional Autónoma de México, 04510 CP, DF, Mexico; ^5^Centro Internacional de Ciencias, Cuernavaca, Morelos, Mexico

## Abstract

Recently, Trifonov's group proposed a 10-mer DNA motif YYYYYRRRRR as a solution of the long-standing problem of sequence-based nucleosome positioning. To test whether this generic decamer represents a biological meaningful signal, we compare the distribution of this motif in primates and Archaea, which are known to contain nucleosomes, and in Eubacteria, which do not possess nucleosomes. The distribution of the motif is analyzed by the mutual information function (MIF) with a shifted version of itself (MIF profile). We found common features in the patterns of this generic decamer on MIF profiles among primate species, and interestingly we found conspicuous but dissimilar MIF profiles for each Archaea tested. The overall MIF profiles for each chromosome in each primate species also follow a similar pattern. Trifonov's generic decamer may be a highly conserved motif for the nucleosome positioning, but we argue that this is not the only motif. The distribution of this generic decamer exhibits previously unidentified periodicities, which are associated to highly repetitive sequences in the genome. *Alu* repetitive elements contribute to the most fundamental structure of nucleosome positioning in higher Eukaryotes. In some regions of primate chromosomes, the distribution of the decamer shows symmetrical patterns including inverted repeats.

## 1. Introduction

It is generally accepted that the chromatin organization of eukaryotic DNA is strongly governed by a code inherent to the DNA sequence. Modulating the accessibility of individual DNA sequences involves many complex interactions, the most prevalent of which are the interactions between histone octamers and DNA in compacted chromosomes [1, 2]. The condensation of DNA into an ordered chromatin structure allows the cell to solve the topological problems associated with storing huge amount of information of chromosomal DNA within the nucleus. In Eukaryotes and Archaea, DNA is packaged into chromatin in orderly repetitive protein-DNA complexes called nucleosomes. Each nucleosome consists of approximately 146-147 bp of dsDNA wound 1.7-1.8 times around a histone octamer [3–5] to form the basic unit of chromatin structure, the nucleosome. Each octamer is composed of two H3-H4 histone dimers bridged together as a stable tetramer that is flanked by two separate H2A-H2B dimers [6]. Stretches of DNA called linker up to 100 bp, often with an increment of 10 bp, separate adjacent nucleosomes. Multiple nuclear proteins bind to this linker region, some of which may be responsible for the ordered wrapping of strings of nucleosomes into higher-order chromatin structures [7].

 Histone proteins condense DNA into complex nucleosome structures both in Eukaryotes and Archaea [2, 8]. Nucleosomes were originally regarded as a distinguishing feature of Eukaryotes prior to identification of histone orthologs in Archaea [9, 10]. The underlying DNA sequence, sometimes called “nucleosome core sequence” or “nucleosome positioning sequence,” acts to bias its own packaging in nucleosomes through preferential positioning of histone octamer. It can facilitate DNA wrapping by placing AA dinucleotides along the portion of the DNA helix that faces the histone core complex [11–13]. Thus, DNA sequences that favor nucleosome formation are enriched with AA dinucleotides spaced ~10 bp apart, resulting in a deficiency of TT dinucleotides at the same location and on the strand facing the histone [11–14]. Five to six nucleotides in either direction, where the complementary strand faces the histone core, the trend is reversed (TT enrichment and a deficit of AA). Two main classes of nucleosome positioning sequence (NPS) patterns have been described. In the first class, AA, TT, and other WW dinucleotides (W = A or T) tend to occur together (in phase) in the major groove of DNA closest to the histone octamer surface, while SS dinucleotides (S = G or C) are predominantly positioned in the major groove facing outward. In the second class, AA and TT are structurally separated (AA backbone near the histone octamer and TT backbone further away), but grouped with other RR (where R is purine A or G) and YY (where Y is pyrimidine C or T) dinucleotides. As a result, the RR/YY pattern includes counterphase AA/TT distributions [15]. 

 In the literature, nucleosome positioning is widely regarded as being sequence specific, enabling them with features of regulation of the access of nonhistone proteins to DNA *in vivo* (e.g., [16]). Albeit, the sequence-dependency of nucleosome positioning is still under debate (see, e.g., [16–21]), the fact that histone proteins in Eukaryotes are highly conserved whereas the genome sequences and the positioning sequence motifs seem to be highly divergent among organisms opens an intriguing question.

Both DNA sequence and nucleosome positioning are important factors in gene regulation [22–24]. Accessibility of transcription binding sites crucially depends on the nucleosome positioning [25, 26]. Nucleosomes are distributed in a highly nonrandom fashion around transcription start sites [27, 28]. Replication is dependent on nucleosome positioning [29].

 Yet the so-called chromatin code has not been fully determined. This code is a well hidden, weak periodical DNA sequence pattern that is recognized by histone octamers. However, the weak signal is not a problem for the histone octamer. It may select the best bendable segments in random DNA sequences. Additionally, as experimental nucleosome mapping indicates, most of the nucleosomes have only marginal stability [13, 29]. It does not mean, however, that their positions are fully uncertain [30, 31]—as much as 50% contribution may come from sequence itself to determine whether a region is covered by a nucleosome or not [16].

The original assumption that DNA sequence is the major factor in nucleosome positioning was first made as early as 1975 [32] and later in 1984 [33] and confirmed afterwards [34, 35]. However, the exact formulation of the positioning pattern remained elusive. Recently, Trifonov's group has provided a pattern that they claim to be an ultimate solution of the long-standing problem of sequence-based nucleosome positioning [36]. Two basic binary periodical patterns are well established: in purine/pyrimidine alphabet—YRRRRRYYYYYR and in strong/weak alphabet—SWWWWWSSSSSW (S/W). Their merger (shifted by 5 bases) in four-letter alphabet sequence coincides with the first complete matrix of nucleosome DNA bendability [37], which was derived from a large database of nucleosome core DNA sequences generated by micrococcal nuclease (MNase) digestion of *C. elegans *chromatin [38, 39]. The results from the bendability analysis indicate that the sequence CGGAAATTTC, called a CG/AT motif, with CG and AT elements 5 bases apart, is predominant in nucleosome cores at the centers of complementary symmetry of the consensus nucleosome-binding pattern derived from bendability data. A more inclusive, but consistent with all previous proposals, consensus nucleosome positioning pattern observed in *C. elegans* was (YYYYYRRRRR)_*n*_. Note that on the reverse complementary strand, the motif is still YYYYYRRRRR (Y/R), but if shifted by 5 bases, it becomes RRRRRYYYYY (R/Y) [40].

 The solution was claimed by Trifonov's group to be unique, hence universal, since the physics of DNA bendability should, in principle, be the same for all species [36]. The simple higher occurrence common consensus of the motifs is TTTCCGGAAA, which is identical to their CG/AT motif derived from *C. elegans* nucleosomes [25, 41]. None of other suggested motifs scores better when compared to the rest of the set. Indeed, the experimental data on *C. elegans* were convincingly consistent with the decamer YYYYYRRRRR in regard to its association to nucleosome positioning partly because the motif was derived from the *C. elegans *MNase digestion data. This alone is a good reason to believe that the CG/AT sequence, as well as the more general YYYYYRRRRR motif, is a universal DNA bendability pattern. Another reason is that this motif can be derived from simple DNA deformability considerations, by minimizing unstacking of bases and base pairs caused by DNA bending on the surface of the histone decamer [36]. 

Analysis of periodicities in 13 fully sequenced eukaryotic genomes [42] showed that weakly periodically positioned TA dinucleotides are detected only in *Saccharomyces cerevisiae*.

 The rationale of our work is as follows. If the generic decamer possesses inherent stability properties making it a universal nucleosome positioning sequence throughout Eukarya, we hypothesized that this decamer signal, caused by a regular spacing of nucleosomes, could also be detected in Archaea, whereby vestiges of primitive nucleosome structures could be identified [10, 43], but lacking in Eubacteria where the nucleosome structure does not exist. The goal of this work was to test the universality hypothesis of the putative nucleosome motif YYYYYRRRRR. To this end, we used mutual information function (MIF) profiles of the generic decamer YYYYYRRRRR along the entire genomes of 3 primate species and 4 species of Archaea. We also tested the S/W motif in all organisms. We show that the overall MIF profiles for the Y/R decamer for each chromosome in each primate species followed a similar periodic pattern, whereas the S/W motif is regular but only in a few chromosomes of primate species. In Archaea species the MIF profiles were different but showed conspicuous periodic features. Hence, with the assumption that an appropriate periodic signal is an indication of the regular spacing of nucleosomes, the Y/R decamer seems to be a highly conserved motif of nucleosome positioning. We used as controls genomes of 3 bacteria, in which there are no nucleosomes, to show that the periodic signal is absent.

On the other hand, the long distance of the regular spacing reflects a low density of the Y/R decamer in these genomes. One implication is that the decamer may not occupy positions at every helix turn, more likely at every nucleosome. Another implication is that other motifs beside this decamer may play a role in the nucleosome positioning.

To further test whether decamer Y/R was able to cast light upon the nucleosome positioning, we generated 10 random sequences of decamers preserving the 5 Ys and 5 Rs content for each chromosome. We found that the random decamers did not present clear-cut patterns in the MIF profiles along chromosomes in contrast to Trifonov's decamer. 

 Our work is consistent with the assumption that Trifonov's generic decamer is one of the nucleosome positioning motifs in primates and in Archaea, and nucleosomes are regularly spaced. However, this motif was derived by conditioning on CG (or AA, AT, TT) as the flanking dinucleotide with periodicity of 10 (CG-8-CG, or AA-8-AA, etc.), which excludes any nucleosome positioning motifs that do not have these periodicities—10 to start with. There may be other motifs that may be associated to nucleosome positioning. This statement comes from our observation that Trifonov's decamer is not found with the same frequencies along different regions of a given chromosome, different local regions within a gene, or GC-rich versus GC-poor segments, even when the DNA is indeed uniformly supercoiled. Actually, there are long stretches in which the generic decamer is absent.

For comparison purposes and for validation of the use of the MIF, here we also report the same analysis in the five chromosomes of* C. elegans*, for which experimental data are available and certain results are expected [41]. With our approach we found that this motif not only reflects well-known periodicities of the nucleosome positions but also there seems to be other previously unidentified periodicities both in primates and Archaea. We conclude that Trifonov's decamer is not the “one-and-only” universal nucleosome positioning motif. We give evidence that these periodicities are associated with highly repetitive sequences in primate genomes. In particular, we show that the Y/R motif is clearly associated to *Alu* repetitive elements in primate species.

## 2. Materials and Methods

### 2.1. Data Sources

 Human (*Homo sapiens*), chimpanzee (*Pan troglodytes*), and macaque (*Macaca mulatta*) complete genome sequences were downloaded from NCBI released, respectively, in March, October, and June of 2006 from ftp://ftp.ncbi.nih.gov/genomes/. In particular, the whole genomes of human, chimpanzee, and rhesus macaque were downloaded from: ftp://ftp.ncbi.nih.gov/genomes/H_sapiens/; ftp://ftp.ncbi.nih.gov/genomes/Pan_troglodytes/, and ftp://ftp.ncbi.nih.gov/genomes/Macaca_mulatta/, respectively.

We selected the following Archaea which were also downloaded from the NCBI website: *Methanocaldococcus jannaschii, Sulfolobus solfataricus, Nanoarchaeum equitans, Archaeoglobus fulgidus, *with the corresponding accession numbers: NC_000909, NC_002754, NC_005213, NC_00917. The selected Eubacteria used as controls are: *Escherichia coli, Pseudomonas fluorescens*, and *Deinococcus radiodurans R1 *with accession numbers: NC_000913, NC_004129, and NC_001263.1, respectively.

### 2.2. An Overview of the Mutual Information Function

 Initially the mutual information (MI) was used to measure the difference between the average uncertainty in the input of an information channel before and after the outputs were received [44]. The MI is a general measure of correlation between discrete variables, analogous to the Pearson product-moment correlation coefficient for continuous variables. For symbolic sequences, MI between two symbols separated by a distance *k* is a function of *k*, called mutual information function (MIF) [45]. The MIF is particularly useful for analyzing correlation properties of symbolic sequences [45].

 Let us denote by *A* = {*a*, *t*, *g*, *c*} an alphabet and by *s* = (…, *a*_0_, *a*_1_,…) an infinite string with *a*_*i*_ ∈ *A*, *i* ∈ *ℤ*, where *ℤ* represents the set of all integer numbers and the values of *a*_*i*_ can be repeated. The MIF of the string *s*  and an identical string shifted *k* positions upstream is defined as

(1) $$\begin{array}{c} I\left(k,s\right)=\sum_{\alpha \in A}\;\sum_{\beta \in A}P_{\alpha ,\beta }\left(k,s\right)\text{lo}{\text{g}}_{2}\left[\frac{P_{\alpha ,\beta }\left(k,s\right)}{P_{\alpha }\left(s\right)P_{\beta }\left(s\right)}\right], \end{array}$$

where *P*_*α*,*β*_(*k*, *s*) is the joint probability of having the symbol *α* followed *k* sites away by the symbol *β* on the string *s* and *P*_*α*_(*s*) and *P*_*β*_(*s*) are the marginal probabilities of finding *α* or *β* in the string *s*. By choosing the logarithm in base 2, *I*(*k*, *s*) is measured in bits. Both the joint probability and the marginal probabilities are estimated throughout the sequence as a global property. The function *I*(*k*, *s*) can be interpreted as the average information over all positions that one can obtain about the actual value of a certain position in the string, given that one knows the actual value of the position *k*-characters away. The mutual information vanishes if, and only if, the events are statistically independent, that is, if all 16 joint probabilities *P*_*α*,*β*_(*k*, *s*) factorize. Thus, the MIF is a function capable of detecting any deviation from statistical independence. It must be noted from (1) that *I*(*k*, *s*) ≥ 0. Computing the MIF for a given sequence using different shifts of magnitude *k* provides an autocorrelation profile. 

### 2.3. The MIF Profile

**Figure 1:**
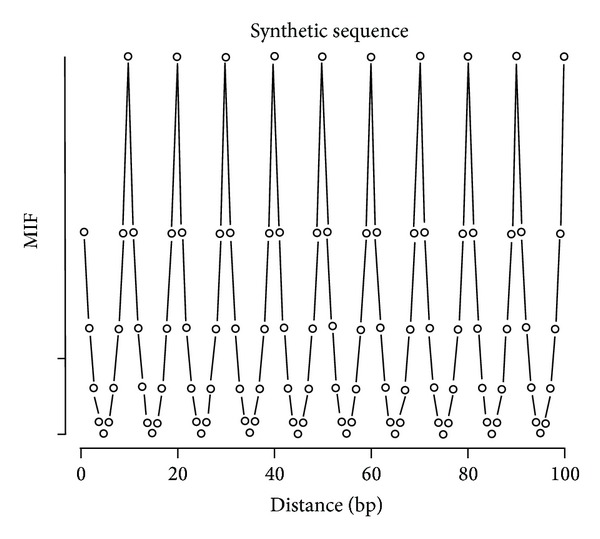
MIF profile of the decamer YYYYYRRRRR from a synthetic sequence. Note the 10-base periodicity.

 In this work, we calculated for each given sequence  *s* the contribution made to *I*(*k*, *s*) by the generic decamers YYYYYRRRRR and SWWWWWSSSSSW. For this purpose, we computed *I*(*k*, *s*) of the sequence  *s* and then marked *s* such that each occurrence of, say, the decamer YYYYYRRRRR appeared in upper case, thereby extending the alphabet to *A*′ = {*a*, *t*, *g*, *c*, *A*, *T*, *G*, *C*}. If we call this marked sequence *s*′, then the difference *I*(*k*, *s*′) − *I*(*k*, *s*) is a measure of how much additional information the decamer YYYYYRRRRR contributes to our prediction of the content of a position in the sequence *k* spaces away from a position whose information content is already known. This renders a brief description of how much of the correlations of a given chromosome are due specifically to the occurrences of the decamer YYYYYRRRRR. MIF, being similar to the autocorrelation function, is a method to detect periodicity in a sequence. A peak in MIF at spacing *k* indicates that the decamer prefers a spacing of *k* bases. In order to test our MIF profile, we generated a synthetic DNA sequence in which the decamer YYYYYRRRRR was placed at regular intervals (Figure 1). Note that the MIF profile clearly exhibits a 10-base periodicity.

For each chromosome the MIF was computed for *k* between 1 and 500. Besides this excess mutual information between symbol and symbol (base and base *k*-position away), an alternative measure of the decamer-decamer correlation is to convert a DNA sequence to a binary (0/1) sequence: 1 for an appearance of YYYYYRRRRR, 0 otherwise. These two methods lead to equivalent results.

Since tandem repeats of YYYYYRRRRR leads to periodicities at *k* = 10, 20,…, in our MIF and since regular spacing of nucleosomes (e.g., 146 bp plus a 45 linker length corresponds to a spacing of 191 bp) leads to periodicity at, for example, 191, 382,…, any periodicities at short (<150) and intermediate (>150 and <400) distances in MIF may indirectly confirm the role of YYYYYRRRRR in nucleosome positioning. 

 This strategy is played out at several levels; we expect to see a periodic presence (absence) of peaks in the MIF profile for genomes known to possess nucleosomes (in those known to have no nucleosomes). We expect to see peaks at both short and intermediate distances. Finally, any observations in contrast to our expectation may lead to new insight; for example, the absence of peaks when expected may point to other nucleosome positioning motifs not included in YYYYYRRRRR; or presence of peaks at unexpected distances may point to other roles of the YYYYYRRRRR motif. 

## 3. Results

### 3.1. MIF Profiles of *C. elegans*

**Figure 2:**
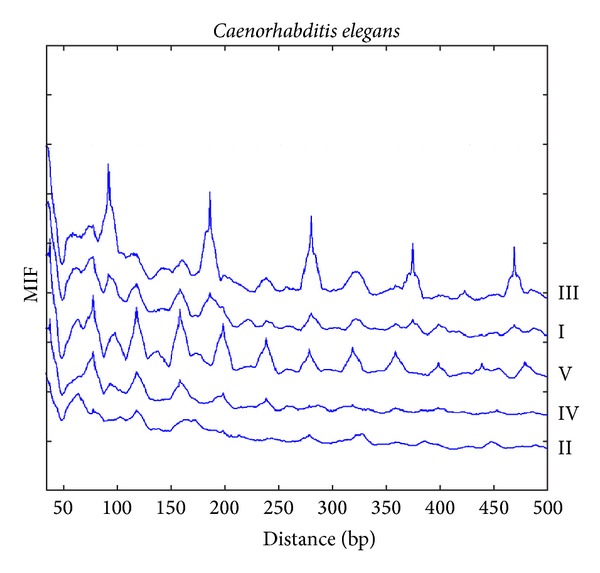
The MIF profiles of the decamer YYYYYRRRRR in 5 *C. elegans *chromosomes.

 Since the decamer was derived from the *C. elegans *MNase digestion data, we expect periodicities to be present in the MIF profile, either due to the tandem repeats of the decamer or due to the regular spacing of the nucleosomes. The MIF profiles of the decamer on chromosomes I, III, and V, but not on chromosome II or IV, of *C. elegans* display a regular pattern of peaks that appear every 10, 20, 40, and 92–94 bp approximately (Figure 2), and they correspond to distance histograms (not shown). This pattern is even met by chromosome X (not shown). The MIF profile of chromosome V shows regular spacings of multiples of 20 bp (e.g., at 120, 160, 200, 240, 320, 360 400, and 480). Given a decamer, we would have expected that bumps (a lumped region like the top of a mountain different from an acute peak) would have a length of 10 bp but what we observed in both primates and *C. elegans* is that the larger the bumps the more repetitions of the decamer in those regions. The different patterns of the MIF profile observed in *C. elegans* imply that nucleosomes do not favor a universal structure even among chromosomes of the same species.

### 3.2. MIF Profiles of *Homo Sapiens*

**Figure 3:**
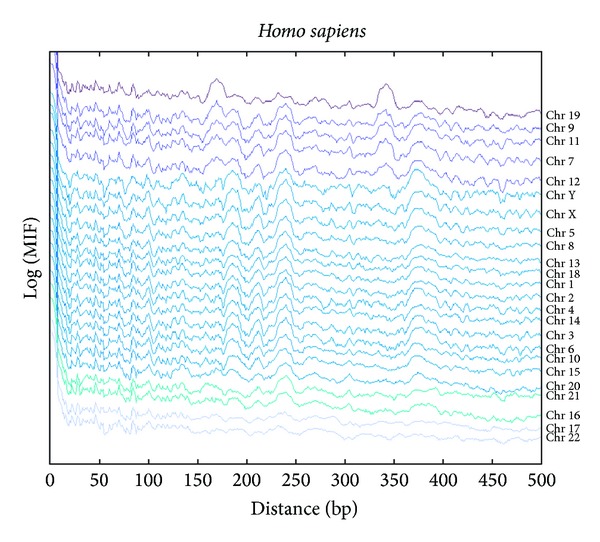
The MIF profiles of the decamer YYYYYRRRRR in all *Homo sapiens *chromosomes.

 In Figure 3, the MIF profiles of the decamer YYYYYRRRRR, for each human chromosome, are illustrated. These profiles, equivalent to correlation functions, correspond to the distribution of spacings between the generic decamer suggested to be associated to the nucleosome positioning. In general, they show rugged landscapes with several troughs (these spacings are avoided) and peaks (these spacings are preferred). The MIF profiles of the decamers on the chromosomes were ordered in order to determine how similar (or different) they are among them. Each MIF profile was shifted upwards by successive integer multiples of 0.5 to facilitate visual inspection. At first glance, there seems to be 3 classes of profiles: class 1 comprises chromosomes 1 to 21 (except 17 and 19) and the sex chromosomes X and Y; class 2 includes chromosomes 17 and 22; and class 3 is represented by chromosome 19. Class 1 can still be subdivided into class 1a (chromosomes 1 to 21 excluding class 1b) and 1b (chromosomes 7, 9, 11, and 12), where the latter displays a bump at around 340 bp and two bumps in the range of 150 to 200 bp. It is widely recognized that the nucleosome has peaks at 80, 146, 165–167, and at around 240 bp [46]. 

A series of peaks up to −162 bp are clearly found in all chromosomes with the use of the MIF. At 10 bp, all chromosomes do not display a peak but they show a deviation in the falling trend. The observed periodicities occur at 31, 47, 62, 72, 84, 103, 110, 132, 136, and 162; bumps occur in regions 180–195, 225–255, and 365–395; long-range periodicities are found at 212, 240, 306, and 345.

**Figure 4:**
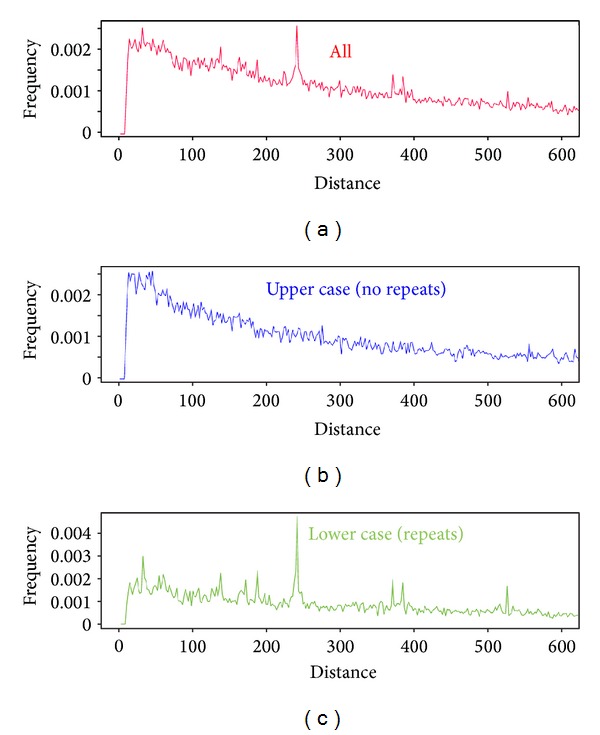
(a) Histogram of the spacing of the decamer YYYYYRRRRR in chromosome 21 of* Homo sapiens* (b) Histogram of the intact chromosome without repetitive elements; (c) Histogram of repetitive elements only.

Most chromosomes display a small peak at 165–167 bp, or 190 bp or 218 bp, which may reflect the periodic spacing between nucleosomes. With the exception of chromosomes 17, 19, and 22, all show a bump at around 240 bp due to repetitive elements as we will shortly illustrate (Figure 4). In addition to these peaks or hills, there are others like the ones found at 345 and 380, which might be considered as the spacing between next-nearest-neighbor nucleosomes. 

This pattern from MIF is consistent with a direct measurement of histograms of the frequency distribution of spacing between the decamer along each chromosome except for the 10-base periodicity. The periodicities observed in the histogram occur at 10, 16, 20, 42, 55, 79, 93, 127, 146, 161, 178, 215, 230, 268, 287, 330, 360, 378, and 472 (not shown). Note that there is a great density of decamers at distances less than 500 bp, and at the same time there are specific peaks directly related to the more conspicuous ones of the MIF profile differences at these distances (Figure 3). 

As close to 50% of the human genome consists of repetitive sequences, and we examine its contributions to the peaks seen in Figure 3. Figure 4 shows the histogram of spacing between the nearest decamer motifs for human chromosome 21 (Figure 4(a)), after masking all repetitive elements (Figure 4(b)), and finally, after masking all the sequence except repetitive elements (Figure 4(c)). A similar behavior is also seen in other human chromosomes (results not shown). Most peaks of intact chromosomes appear also in the histogram of repetitive sequences only. From Figure 4, it can be seen that the histogram of spacing of decamer YYYYYRRRRR for *H. sapiens* chromosome 21 shows peaks that appear in both the whole genome (Figure 4(a)) and in the only repetitive sequences (Figure 4(c)). In particular, this is true for the 240-241 peak. This means that in repetitive sequences there is a great deal of consecutive occurrences of the YYYYYRRRRR decamer spaced 240-241 bp apart. The biological meaning of this observation is still unknown. Due to the large proportion of repetitive sequences in the human genome, its potential function cannot be ignored. Our findings, as well as those in [46, 47], point to a potential role of repetitive elements in the nucleosome positioning. In Supplementary Information S1 available online at http://dx.doi.org/10.1155/2013/963956, we show a table of spacings between YYYYYRRRRR found at highly repetitive sequences in the human genomes.

**Figure 5:**
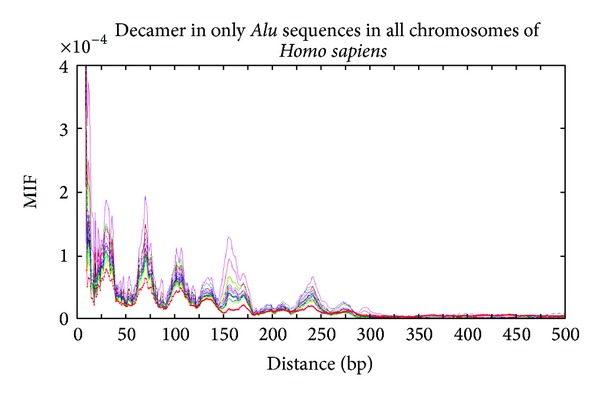
MIF profiles of the R/Y decamer in only *Alu *sequences in all *Homo sapiens* chromosomes.

A possible relation between nucleosome positioning and one particular type of repetitive sequences, the *Alu* elements, has been suggested before [46]. It was observed that if one ignores the *Alu* repeats, several peaks in the Fourier spectra for AA/TT sequence (1 for AA or TT, 0 otherwise) disappear, but some peaks like the one found at about 165 bp still linger [46]. A similar observation was reported in [36]. Note that here we are analyzing a very different sequence (1 for YYYYYRRRRR, 0 otherwise), and repetitive sequences besides *Alu* are also masked. When only *Alu* sequences are considered, the MIF profiles of the decamer R/Y in all human chromosomes (Figure 5) display the same pattern in all chromosomes, indicating a strong association between the decamer Y/R (and R/Y) with *Alu* sequences. There are pronounced peaks at 32, 62, 110, 134, 160, and at 240 in all human chromosomes. There is a slight departure of this pattern in chromosome X (red curve).

**Figure 6:**
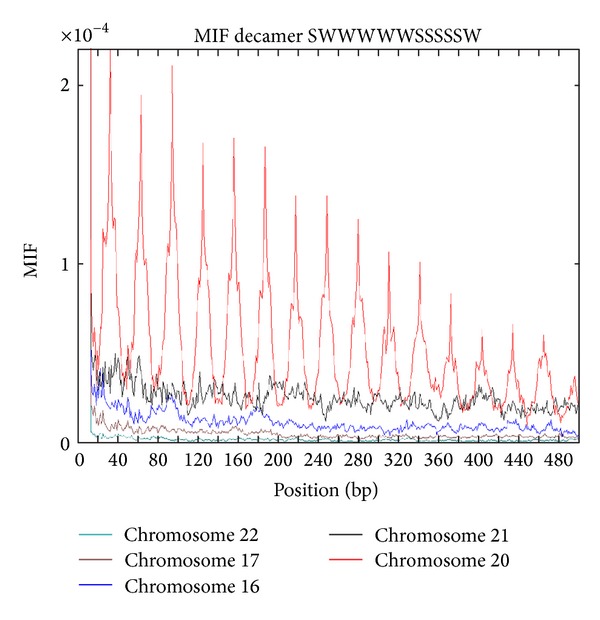
The MIF profiles of the 12-mer SWWWWWSSSSSW in some *Homo sapiens *chromosomes.

When the spacing of more specific decamers of the type YYYYYRRRRR (e.g., CGGAAATTTCCG) is analyzed, the periodic signal weakens considerably. The MIF profiles of the 12-mer SWWWWWSSSSSW in some chromosomes of *H. sapiens* are shown in Figure 6. Note that there is a regular behavior only in chromosome 20 in which there are peaks every 30 bp. A regular behavior is also observed in chromosome 12 whereas the remaining chromosomes exhibit a more irregular and nonuniform pattern.

**Figure 7:**
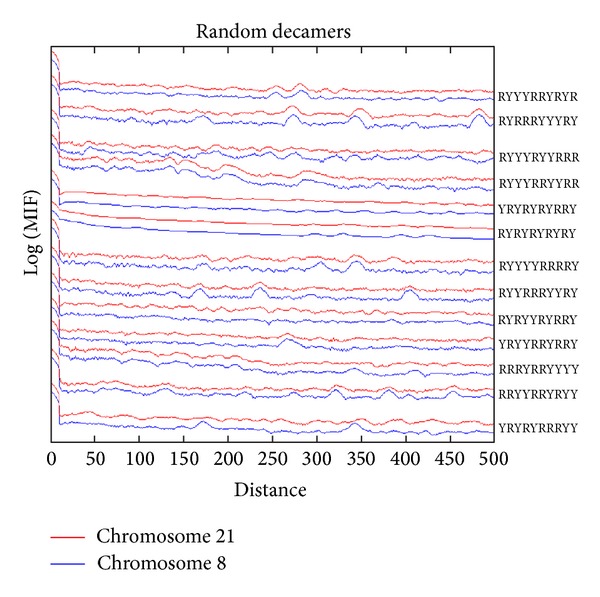
MIF profiles of random decamers with 5 purines and 5 pyrimidines along *Homo sapiens* chromosome 21, in order to compare the meaningful signal of YYYYYRRRRR as a binder nucleosome motif.

If one selects at random a given decamer (preserving the number of Ys and Rs), not surprisingly, in most cases no prominent periodic signals are found as it is illustrated for the two chromosomes 21 and 8 of *H. sapiens *in Figure 7. The MIF profiles of the controls were not statistically similar among them (average correlation coefficient *r* ^2^ = 0.56) as the generic decamer in intact chromosomes do (*r* ^2^ = 0.76). The average correlation between the actual chromosome 8 with all random controls was *r* ^2^ = 0.42 whereas the average correlation between chromosome 21 with all random controls was *r* ^2^ = 0.65. Note that in the intact chromosomes we preserve the YYYYYRRRRR content and in the shuffled control we respect the nucleotide content but we disrupt the YYYYYRRRRR sequence. Therefore, the MIF profiles of the controls were not similar among chromosomes as the generic decamer do in intact DNA sequences.

### 3.3. Nonhuman Primate MIF Profiles

**Figure 8:**
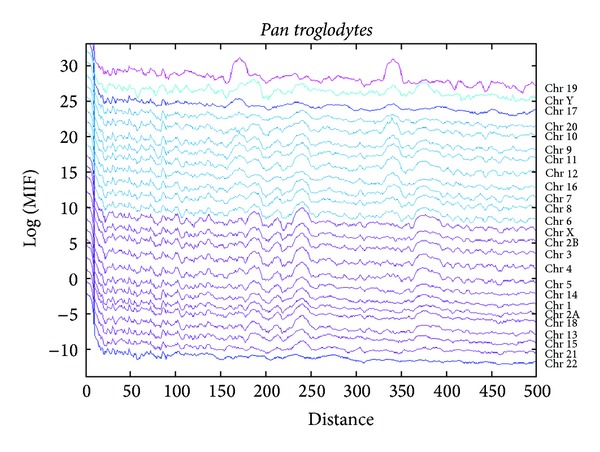
The MIF profiles of the decamer YYYYYRRRRR in all *Pan troglodytes *chromosomes.

**Figure 9:**
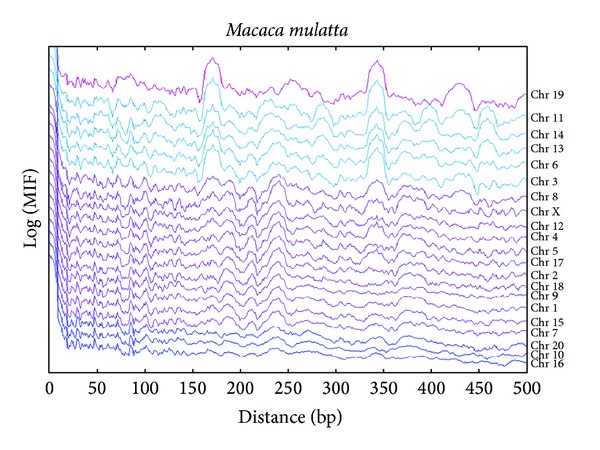
The MIF profiles of the decamer YYYYYRRRRR in all *Macaca mulatta *chromosomes.

 We also calculated the MIF profiles of the decamer on all available chromosomes of *Pan troglodytes* and *Macaca mulatta *(Figures 8 and 9). There is a consistency between MIF profiles of all chromosomes for each primate species, even though subtle differences exist. However, a more striking finding is that when two species are compared, a MIF profile for the decamer on a chromosome of a given species is more similar to that on the same chromosome but in the other two species, than to the MIF profile on different chromosomes of the same species. 

In general, it is clear that despite the different evolutionary histories of the 3 primate species, there is a common pattern in the MIF profiles of the generic decamer on a given chromosome.

 For the same comparative purposes, the MIF profiles of the decamer on the chromosomes of *P. troglodytes* (Figure 8) can also be divided into the same three classes in which the *H. sapiens* chromosomes were divided. In the first class, there are chromosomes 1 to 21 and the sex chromosomes X and Y; in class 2, chromosomes 17 and 22 can even be subdivided given a conspicuous widening similar to a bump in the range of 150 to 175 that is present in chromosome 17. Class 3 is also represented by chromosome 19. But the first class can be subdivided into class 1a (chromosomes 1 to 21 excluding class 1b and 1c), class 1b with the same characterization that is, in human MIF profile (chromosomes 6 to 12, 16 and 22), and class 1c is represented by chromosome Y which has a different kind of bumps in the range of 64–84 bp and in the range of 164–200 bp (Figure 8).

The MIF profiles of the decamer on all chromosomes analyzed in *M. mulatta *(Figure 9) seem to pertain to only two classes. Class 1 can be subdivided by shorter amplitudes in the same bp signals between class 1a (chromosomes 10, 16, and 20) and class 1b (chromosomes 1, 2, 4, 5, 7 to 9, 12, 15, 17, and 18). With a similar profile than the two mentioned subclasses, class 1c presents highly conspicuous peaks at around 172 and 342 bp, and a bump in the range of 274 to 300 bp (chromosomes 3, 6, 11, 13, and 14). In class 1c, the chromosomes 11, 13 and 14 also have a peak at around 460 bp.

 Class 2 is represented by chromosome 19 that, in contrast to chromosome 19 for *P. troglodytes* and *H. sapiens,* has a bump in the range of 400 to 450 bp which it shares only with chromosomes 3, 8, and 11, beside the features of its own profiles class (Figure 8). It is important to note that there are several common peaks among human, chimpanzee, and rhesus macaque at 31, 47, 62, 72, 84, 103, 110, 132, 136, and 162; even some bumps are shared among the three species at 180–195, 225–255, and 365–395 and some long-range periodicities at: 212, 240, 306, and 345.

It is important to mention that considering an alphabet of *A* = {*A*, *T*, *G*, *C*}, we calculated the MIF for the 3 species of primates masking all repeats (not shown) and all peaks disappear.

 We estimated the similarities of the chromosomes within and between species based upon the cross-correlations of the MIF profiles of the chromosomes for the 3 primate species. All Pearson's correlation coefficients within chromosomes of a given primate species display values which are in a rough agreement with the classes mentioned above (see correlations in S2). The Pearson correlation coefficients of chromosomes between the 3 primate species in general also support our visual inspection of the previous observations of heterogeneity between chromosomes within species, and uniformity among the same chromosome between species (see S2).

 In general, it is clear that despite the subtle differences among chromosomes within species, there is a common pattern in the MIF profiles of the decamer. Given that this decamer is a consensus motif for nucleosome positioning sequence, the hypothesis that the statistical properties of the decamer can be translated to those of the nucleosome positioning can be put forward.

**Figure 10:**
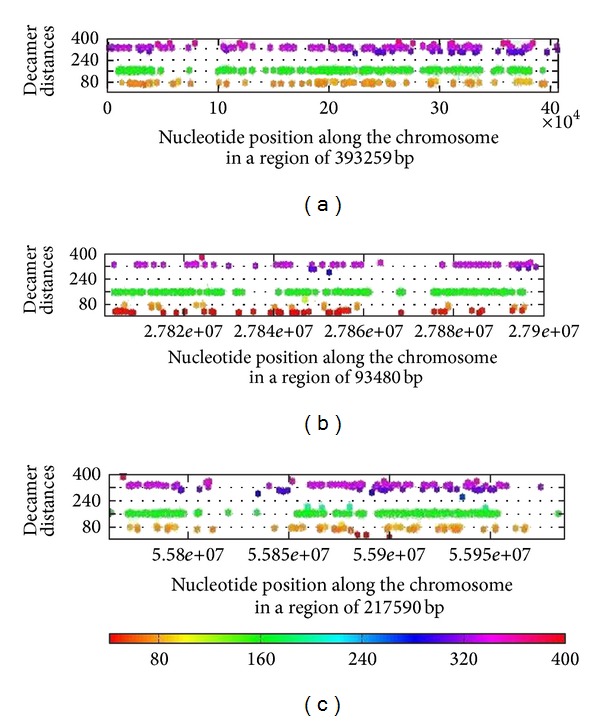
The three main regions of human chromosome 19, where distances around 80, 160, and 320 between the generic decamer are more highly concentrated than in the rest of the chromosomes are highlighted. Note that the clusters correspond to the peaks observed in their respective MIF profiles.

The distribution of the decamer YYYYYRRRRR along each chromosome is not uniform since there are regions in which clusters are crisply recognized whereas there are long stretches lacking this decamer (Figure 10). Since MIF and spacing histograms are averaged over all regions in a chromosome, Figures 3–10 do not show the heterogeneity information in regions deserted of this decamer. Therefore, other decamers or signals associated to the fine structure of the chromosomes cannot be ruled out.

**Figure 11:**
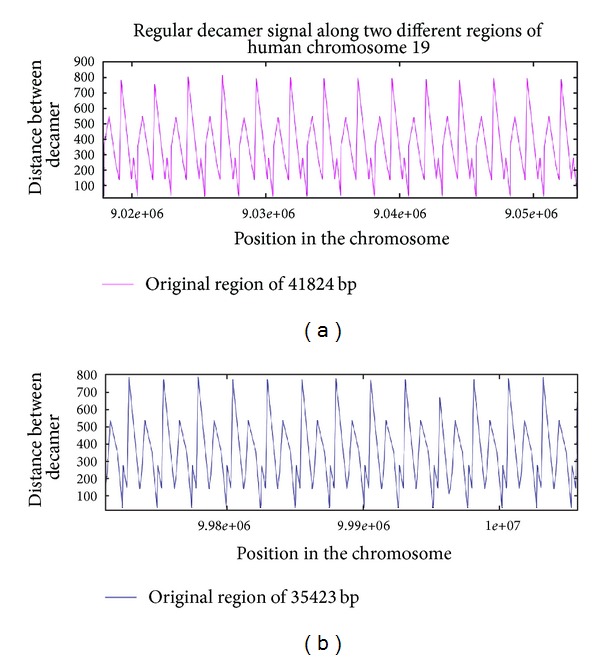
Plot of the distances between the generic decamer (ordinate) along two regions of chromosome 19 (abscissa) of *Homo sapiens*. Note the inverted repeat sequence.

 To further examine the issue of heterogeneity, we show examples of physical maps of the location of the generic decamer along a given chromosome. In Figures 10 and 11, the location of the generic decamer along chromosome 19 of *H. sapiens *for different magnification scales is shown. The most striking observation is that the decamer positions are not random but they are not uniformly distributed along the chromosome either. The decamer distribution is clumped in certain regions but there are long stretches in which the decamer is plainly absent. For the remaining human chromosomes, nucleosomes are also more consistently positioned than expected by chance and many are organized in regularly spaced arrays that are enriched near active chromatin. Hence, nucleosome positions are also clearly influenced by DNA sequence. A striking example is an array of regularly spaced nucleosomes created by tandem repetition of sequences with strong nucleosome positioning properties across approximately 35,423 and 41,824 bp of chromosome 19 (Figures 10 and 11). Similar arrays can also be found in other chromosomes.

If we take a look at the distances between consecutive appearances of the decamer, there are regions of the chromosomes in which the decamer appear in a periodic manner. That is, there are two (or actually more) stretches of the chromosome which contain the same number of this decamer, but with the peculiarity that the first and second occurrences of the decamer are spaced by the same distance in both stretches, and so are the second and third, and so on (not shown). Another interesting feature is that there are arrangements of different distances in which the order of distances of the generic decamer can also be encountered in some downstream regions but exactly in reverse order of those distances. In other words, the distribution of the decamer exhibits an inverse symmetry (see Figure 11). It is worth mentioning that in this region there are genes of cadherin, beta-catenin and zinc fingers. This is consistent with a recent finding about rare roughly symmetrically positioned nucleosomes such as the zinc-finger containing protein that showed roughly symmetrically positioned nucleosomes [48]. 

### 3.4. MIF Profiles of Archaea and Eubacteria Species

 We examine the MIF profiles of the decamer for the following Archaea species: *Methanocaldococcus jannaschii*, *Archaeoglobus fulgidus*, *Sulfolobus solfataricus*, and *Nanoarcheum equitans*. 

**Figure 12:**
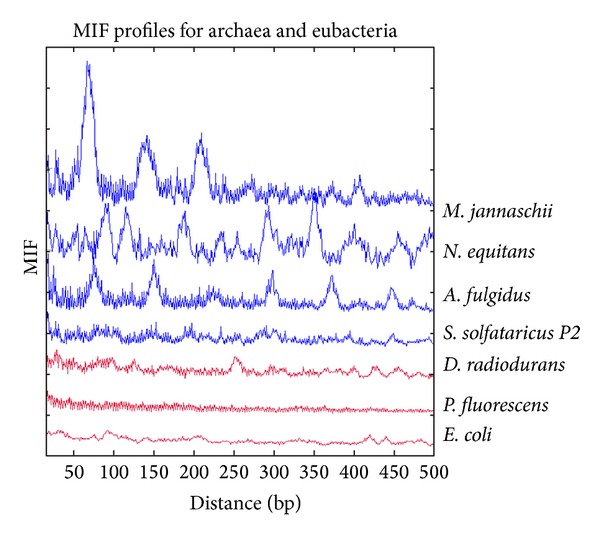
The MIF profiles of the decamer YYYYYRRRRR in some Archaea and Eubacterial genomes.

 In Figure 12, the MIF profiles of the decamer on several Archaea species are illustrated. It is remarkable to observe that this decamer still exhibits conspicuous periodicities. Similar to the MIF profiles observed in primates, in general, the MIF profiles of the decamer in archean species also manifest rugged landscapes with several troughs and peaks. 

 In *M. jannaschii, *there are several prominent peaks at around 67, 141, 210, and 408 bp whose magnitudes decrease with distance and they are interspersed throughout high-frequency oscillatory dynamics. The spacing of 141 apparently matches that of a nucleosome core sequence length and that of 67 close to half that length. The spacing of 210 could match the distance between two neighboring nucleosomes, and 375 for next-nearest-neighbor nucleosomes. As various linker sequence length may coexist in different regions in the genome, two nucleosome spacings (375/2 = 187.5 and 210) may not necessarily contradict each other. 

 In* N. equitans, *there are several salient peaks at around 27, 89, 93, 115, 189, 234, 294, 352, 408, 456, and 496 bp with a great variability in their magnitudes, and they are embedded in a high-frequency oscillatory behavior. 

Note that in *M. jannaschii* and *N. equitans *there are peaks at ~60 bp and ~85 bp as were found in pLITMUS28 and in *Methanothermus fervidus *[49]. Archaea nucleosomes resemble the structure formed by the (H3 + H4)_2_ tetramer at the center of the eukaryotic nucleosome. Both structures have a histone tetramer core that recognizes positioning signals, directly contacts ~60 bp, and wraps ~85 bp of DNA alternatively in either a positive or negative toroidal supercoil [49].

 In *A. fulgidus,* the MIF profile displays a pattern of high-frequency oscillatory structure that they themselves form jagged bumps, and there are salient peaks at around 75, 150, 300, 375, and 450.

 In* S. solfataricus, *the MIF profile is essentially composed by high-frequency oscillatory structure from which jagged bumps are formed with no discernible prominent bumps.

 In order to test whether the MIF profiles of Archaea are biologically meaningful, that is, the periodic appearance of the putative nucleosome positioning decamer is due to the repetitive motif within a nucleosome core and the regular spacing of nucleosomes, we also show the MIF of the decamer for several bacteria which are known to be lacking nucleosomes. Three of them (*Escherichia coli, Pseudomonas fluorescens, *and* Deinococcus radiodurans*) are shown in Figure 12. Note that in the corresponding MIF profiles of these three bacteria, the signal is so weak that we cannot ascribe, as expected, that there is a periodicity of the YYYYYRRRRR decamer along the genome. If the decamer is indeed associated with the nucleosome positioning sequence in any species, this is consistent with the absence of nucleosomes in bacterial genomes. We included these bacteria to test whether Archaea cells show evolutionary selection either for or against sequences that favor nucleosome formation. As bacteria do not possess histones, but do show 3 and 10-11 base periodicity due to coding regions, we presumed that *E. coli, P. fluorescence, *and* D. radiodurans* DNA sequences are evolutionarily neutral with respect to nucleosome formation, such that preferred nucleosome forming sequences will occur by chance. These results strongly argue that the Archaean genomes have evolved to favor nucleosome formation.

## 4. Discussion

In this work, we have found that the proposed nucleosome positioning motif YYYYYRRRRR exhibits expected periodicities in primates and Archaea, thus consistent with the hypothesis that it plays a role in nucleosome positioning. In particular, we placed emphasis on the effect of repetitive sequences on the observed periodicities of the motifs R/Y and Y/R, as well as the S/W motif. We succeeded in the detection of the periodical repetition of the DNA patterns in all chromosomes tested despite weak or previously undetected periodicities with other methods. The extraction of the periodical signals in all chromosomes was due to the fact of using both MIF profiles and the generic decamer R/Y and Y/R to document a comprehensive distribution of nucleosome DNA sequences in primate species and even perhaps in Archaea. The MIF profiles display peaks or bumps in places previously recognized, such as the typical signatures at 31-32, 84, 146, 157, 171 and 200 [25, 41, 46]. New periodicities such as 100, 167, 240, and 320 are reported here. We did find the 10-bp in the histograms (not shown) but not in the MIF profiles because it may be unlikely to detect it. The rationale is as follows: there are 10 million copies of YYYYYRRRRR/RRRRRYYYYY in the human genome and if we assume they do not overlap, this would lead to 90 million bases (when they do overlap, the number would still be smaller). For example, for a segment …YYYYYRRRRRYYYYY… which contains 2 copies of the motif, it covers only 15 bases instead of covering 20 bases; 90 million bases represent 3% of the human genome (if overlap exists, could be 2%), but at least 20% of the human genome is well positioned with nucleosomes. Therefore, there are not enough 10 mers to cover densely within a nucleosome positioning region. This dense packing is what would lead to the periodicity of 10. On the other hand, we can have longer periodicities. Suppose we have this order: beginning-middle (dyad)-end-linker-beginning-next-nucleosome-…. Assume also that this motif tends to sit at the beginning of a nucleosome, then we do not need 20 copies per nucleosome to cover the whole region, only 1-2 copies per nucleosome at the beginning. This density is more consistent with our observations. Then, the regular spacing of nucleosomes would lead to longer (+200) periodicities, but not the 10-base periodicity within a nucleosome. When repetitive elements were masked in whole chromosomes it became evident that the decamer contributes not only to the presence of the nucleosome structure but it also manifests itself as part of highly repetitive sequences (see S1).

With more than one million copies, *Alu* elements are the most abundant repetitive elements in the human genome; they represent ~10% of the genome mass and belong to the SINE (short interspersed elements) family of repetitive elements [50]. *Alu* elements emerged ~55 million years ago from a fusion of the 50 and 30 ends of a 7SL RNA gene, which encodes the RNA moiety of the signal recognition particle (SRP). Modern *Alu* elements are ~300 bp in length and are classified into subfamilies according to their relative ages [51]. Dimeric *Alu* elements are unique to primates. *Alu* RNAs, transcribed from *Alu* elements, are present in the cytosol of primate cells. *Alu* elements inherited the internal A and B boxes of the RNA polymerase III (Pol III) promoter from the 7SL RNA gene [52]. The typical *Alu* RNA is a dimer of related but nonequivalent arms that are joined by an A-rich linker and followed by a short poly(A) tail [52].

Not surprisingly, the MIF profiles of the shuffled decamers showed no discernible pattern and no rugged landscape. The MIF profiles of the controls were not similar among chromosomes as the generic decamer was. The MIF profiles of the generic decamer in the three primate species exhibited a uniformity between species for the same chromosome, but heterogeneity within species between different chromosomes. The observed regularity of the patterns allowed us to provide families for the distribution of the generic decamer tested.

We selected the three densest regions in which there were clearly clusters of the decamer which appeared every 80, 160, and 320 bp (multiples of 80) of human chromosome 19 (Figure 10). These clusters of the decamer clearly correspond to the peaks of the MIF profile of human chromosome 19 (Figure 3).

The finding of regular periodic patterns of the decamer along primate chromosomes visualized in distance series of long stretches of the different chromosomes, as well as the patterns reflecting inverted repeats (inverse symmetry), discards the possibility that the generic decamer is biologically meaningless. Periodicities naturally arise if the decamer is tandemly repeated, and/or if the nucleosomes are regularly spaced. Inverted symmetry can be caused by the central role of dyad in the nucleosome cores. We think that the probability of finding such arrangements just by chance would be very low. The patterns of the MIF profiles of the five chromosomes of *C. elegans* are not entirely consistent with the regular reported structure of their nucleosomes [41]. Therefore, most nucleosomes in primate genomes are consistently positioned, either because they are forced into positioned arrays by chromatin remodeling or DNA binding proteins, and/or because they adopt favored sequence positions in genomic regions without active binding. Interestingly enough, the MIF profiles of the generic decamer in all Archaea tested showed prominent peaks in an oscillatory background. We propose that this decamer deserves further studies in order to determine if it has been selected since the origin of nucleosome structure. 

It has been noted that the RNA motif SRP9/14 binds primarily to the universally conserved core of the *Alu* RNA 59 domain, which forms a U-turn in the context of a tau-junction [53]. This RNA motif is highly conserved in the SRP RNAs from higher eukaryotes to yeast and from Archaea to some Gram-positive Eubacteria [54]. A dimeric *Alu* RNP complex might be important in the origin or propagation of tandemly arranged *Alu *retroposons, as retropositional success was clearly correlated with the emergence of dimeric *Alu* elements during primate evolution [55]. *Alu* elements play an important role in the regulation of gene expression at various levels, such as in alternative splicing when present in intronic regions of genes [56]. The observed MIF profiles from different chromosomes or different species often differ substantially. Therefore, all these patterns cannot be attributable to the origin of nucleosome structures, or nucleosomes sequence preferences. It is likely that many of the peak features may be ascribed to some species-specific or chromosome-specific DNA sequence features, such as *Alu* repeats, but not necessarily limited to them.

What then accounts for the phenotypic differences between nonhuman primates and humans? It stands to reason to propose that part of the difference might be because of species-specific alternative splicing.

We were able to characterize different classes of MIF profiles within each primate species. The outstanding observation is that the MIF profile of a given chromosome is more similar to the corresponding profile among species than within species. The observed peaks of the MIF profiles using the generic decamer in primates are strongly associated with several highly repetitive sequences. This is in agreement with the recent discovery that the positioning of neighboring nucleosomes seems to be in phase with *Alu* elements as reflected by peaks in the Fourier analysis at 84-bp and 167-bp [46]. In this work, we corroborate this result with both Fourier (not shown) and MIF analyses using the decamer.

We have also found that human repetitive sequence densities are mostly negatively correlated with R/Y-based nucleosome-positioning motifs (NPM) and positively correlated with W/S-based motifs [57]. The positive correlation between YYYYYRRRRR/RRRRRYYYYY and repetitive sequence density is intriguing, as it provides an exception to negative correlation between densities of repetitive sequences and that of R/Y-based NPMs. The scatter plot for YYYYYRRRRR/RRRRRYYYYY is particularly interesting; despite the negative trend followed by the majority of the points, there is a minority trend for high repetitive sequence densities and high NPM densities [57]. We believe that it is in this region in which the generic decamer can be found positively associated with *Alu *elements.

Herein, we focused on MIF profiles of the type R/Y and Y/R with several peaks that overlap with repetitive elements. Amongst the most prominent peaks for most chromosomes in primates are at 84, 100, 167, and 240. In fact, in certain chromosomal segments, a well-defined periodic pattern of the decamer within highly repetitive sequences was observed. Appearance of the CG dinucleotide in the nucleosome positioning pattern is rather surprising, considering its generally low occurrence in eukaryotic sequences. However, recent studies suggest that CG dinucleotides play a special role indeed [36]. First, it displays 10.4-base periodicity almost as often as the AA and TT dinucleotides do, in particular in G+C-rich regions [42, 58]. In the *Alu* sequences, the CG element appears at a distance of 31-32 bases from one another [59], suggesting involvement of the sequences in the nucleosomes. Methylation/demethylation of CpG would modulate the nucleosome stability, so that the CG-containing nucleosome could be considered as “epigenetic nucleosomes” [59]. Most chromosomes, except 19, 22, X, and Y, show a notorious similarity in regard to the putative positioning of the nucleosomes as obtained with our approach. Even among species within the primate family, the latter still holds. Hence, the conserved MIF profile on primates can reflect the importance of these generic decamer into the architecture of primate genomes. In addition, the conserved and peculiar organization of islands into repetitive elements may allow us to consider that this specific decamer could be implicated in the self-regulation functions inherent in these types of sequences.

The finding of peaks in Archaea and its absence in Bacteria may not be a surprising result since it is known that the former contain histones whereas the latter do not. But it is noteworthy that we have detected for the first time via the MIF profiles putative nucleosome signals in Archaea. In addition, there is a prominent presence of the generic decamer in Archaea as it is shown in their corresponding histograms (not shown). To our knowledge, this is the first description of this generic decamer for the nucleosome in this group and it remains to prove that it may be considered a nucleosome without the subsequent evolutionary refinements conferred by the repetitive elements. Hence, repetitive elements turn out to be basic ingredients of the most fundamental structure of nucleosome positioning in higher Eukaryotes.

In summary, putative nucleosome positioning motifs (NPM) associated to repetitive elements in human, nonhuman primates, and Archaea have been identified by means of mutual information profiles (MIF). Trifonov's group suggested a most recent “finale motif” of the long-searched “chromatin code.” The biological significance of this decamer motif and its two degenerate parental motifs is examined in primates and Archaea. Common features in the patterns of the generic decamer R/Y on MIF profiles among primate species are found. The distribution of R/Y motif exhibits previously unidentified periodicities, which are associated to highly repetitive sequences in the genome. *Alu* repetitive elements may contribute to the most fundamental structure of nucleosome positioning in higher Eukaryotes. In some regions of primate chromosomes, the distribution of the R/Y decamer shows symmetrical patterns including inverted repeats. We have detected for the first time via the MIF profiles putative nucleosome signals in Archaea. It is clear that the R/Y motif is relevant in the NPM but it is also certain that there must be other relevant motifs besides the Trifonov “finale.” Our findings may contribute to the understanding of the origin of nucleosome structures in Archaea and its remarkable success of *Alu *retroposons in colonizing primate genomes.

## Supplementary Material

Supplementary Information S1 consists of a table of spcacings between YYYYYRRRRR found at highly repetitive sequences in the human genome.
Supplementary Information S2 consists of all Pearson correlation coefficients of the MIF profiles for all the chromosomes for the 3 primate species.
