# Supplementary material for: Periodic Distribution of a Putative Nucleosome Positioning Motif in Human, Nonhuman Primates, and Archaea: Mutual Information Analysis
Source: Int J Genomics. 2013 Jun 10;2013:963956. doi: 10.1155/2013/963956 (PMC3691935; doi:10.1155/2013/963956)
Supplement: Supplementary file 1 — Supplementary Information S1 consists of a table of spcacings between YYYYYRRRRR found at highly repetitive sequences in the human genome. Supplementary Information S2 consists of all Pearson correlation coefficients of the MIF profiles for all the chromosomes for the 3 primate species. [file 963956.f1.pdf]

Supplemenatry Information S1

Spacings between* YYYYYRRRRR *found at highly repetitive sequences in the human genome

Sequences obtained from Repbase (http://www.girinst.org/server/RepBase/index.php)	Spacings between YYYYYRRRRR in each sequence
HERVK9I.ERV2.*Homo.sapiens*	241
MSTB1.ERV3.Primates	240
MER58C.hAT.Eutheria	331,2401,66,692,52,498,28,465
HERVL_40.Endogenous.Retrovirus.Homo.sapiens	240
LTR12E.ERV1.*Homo.sapiens*	240
MLT1F1.ERV3.Eutheria	240
CR1_Mam.CR1.Mammalia	240
MER70_I.LTR.Retrotransposon.Eutheria	240
LTR46.ERV1.*Homo.sapiens*	240, 446
MER121.Transposable.Element.Homo.sapiens	168,109,350,245,961,23,797,1304,1016,289,223,298,318,122,156,241,196,36,629,402,373
Charlie22a.hAT.Mammalia	241
CHARLIE4.hAT.Eutheria	241
LTR83.ERV3.Eutheria	241
MLT1H2.ERV3.*Homo.sapiens*	241
MER2.Mariner/Tc1.Eutheria	415,377,187,100,87,137,1199,32,857,385,371,528,241
MER65I.Endogenous.Retrovirus.Eutheria	143,270,271,536,167,111,14,267,15,271,912,53,321,391,263,714,857,1284,241
MamRep1527.Transposable.Element.Eutheria	256,347,1232,132,770,767,574,782,57,1050,241
SVA2.MSAT.Eutheria	241
MER54A.ERV3.*Homo.sapiens*	241, 536
MER94.hAT.*Homo.sapiens,*	241
KANGA2_A.Mariner/Tc1.*Homo.sapiens*	241
MER34B_I.Endogenous.Retrovirus.*Homo.sapiens*	241
MER50B.ERV1.Homo.sapiens	266,139,36,193,144,31,71,145,127,368,782,419,296,241,230,531,50,71,129,35,944
L2B.CR1.Eutheria	241
LTR36.ERV1.*Homo.sapiens*	241
MSTC.ERV3.Eutheria	241
MER83C.ERV1.*Homo.sapiens*	241

MER21C.ERV3.Eutheria	63,146,176,979,374,137,518,682,32,857,385,899,241
Kanga1.Mariner/Tc1.Mammalia	241
THE1C.ERV3.*Homo.sapiens*	241, 537
MER45C.hAT.*Homo.sapiens*	241
MER80B.hAT.*Homo.sapiens*	241
L1P4d_5end.L1.Primates	241, 519, 584
LTR52.ERV3.*Homo.sapiens*	241
LTR14C.ERV2.*Homo.sapiens*	241
LTR9C.ERV1.*Homo.sapiens*	104,606,1418,496,746,1392,241
L1M3A_5.L1.Eutheria	241
MER101B.ERV1.*Homo.sapiens*	241
LTR10A.ERV1.Eutheria	241, 287
LTR71A.ERV1.*Homo.sapiens*	241
MER54B.ERV3.*Homo.sapiens*	282,548,62,445,383,53,738,977,197,660,187, 1097,241
HERVH48I.Endogenous.Retrovirus.*Homo.sapiens*	241
HERVIP10FH.ERV1.*Homo.sapiens*	241
CHESHIRE_A.hAT.*Homo.sapiens*	241
MER83AI.ERV1.*Homo.sapiens*	241
MER101.ERV1.*Homo.sapiens*	241
LTR21A.ERV1.*Homo.sapiens*	242
ACRO1.SAT.*Homo.sapiens*	54,143, 242, 352
